# Is there a difference in testosterone levels and its regulators in men carrying BRCA mutations?

**DOI:** 10.18632/oncotarget.21802

**Published:** 2017-10-10

**Authors:** Hanan Goldberg, Liat Shavit Grievink, Roy Mano, Yaara Ber, Rachely Ozalbo, Sivan Tuval, Jack Baniel, David Margel

**Affiliations:** ^1^ Division of Urology, Rabin Medical Center, Petah Tikva and The Sackler Faculty of Medicine, Tel Aviv University, Tel Aviv, Israel

**Keywords:** BRCA mutations, free androgen index, luteinizing hormone, prostate cancer, testosterone

## Abstract

**Background:**

Male BRCA mutation carriers are at risk for an early onset aggressive prostate cancer. No data exist on the association of testosterone levels among these patients. We aimed to analyze testosterone and associated hormonal levels among male BRCA carriers and non-carriers.

**Patients and methods:**

Overall 87 male carriers and 43 non-carriers aged 40-70 were prospectively enrolled. Clinical data were collected and all patients were tested for total testosterone (TT), prostate specific antigen (PSA), follicle stimulating hormone (FSH), luteinizing hormone (LH), free androgen index (FAI), sex hormone binding globulin (SHBG) and prolactin. Multivariate linear regression analysis was performed to predict TT levels.

**Results:**

The median age, mean BMI, comorbidities, PSA, FSH, LH and SHBG levels in both groups were similar. However, mean TT and FAI were higher in the carriers (16.7 nmol/l vs 13.5 nmol/l, p=0.03 and 39.5 vs 34.8, p=0.05, respectively), while prolactin was significantly lower. Multivariate analysis demonstrated that while BMI was inversely correlated to TT levels in both groups, LH was a predictor only in non-carriers.

**Conclusions:**

Carriers have higher TT and FAI levels and lower prolactin levels; but LH does not predict their TT levels. Further research in a larger cohort of BRCA carriers with and without prostate cancer should be performed.

## INTRODUCTION

Breast Cancer susceptibility genes 1&2 (BRCA1&2) are involved in DNA repair and cell cycling [1]. Genetic instability is a characteristic of BRCA1& 2 deficient cells that leads to an accumulation of genomic and post genomic abnormalities [1]. Autosomal dominant germ-line mutations in BRCA1&2 among women are associated with a lifetime risk of 85% for breast cancer and 45% for ovarian cancer [2]. In males, mutation carriers are at increased risk of several other cancers including male breast cancer, pancreatic adenocarcinoma, melanoma and prostate cancer (PCa) [3, 4].

Several studies have reported that the risk of PCa is higher among male carriers of BRCA1& 2 mutations [5-8]. The results from the Breast Cancer Linkage Consortium showed a relative risk (RR) of 4.65 of PCa among male BRCA2 mutation carriers with a RR of 7.33 below the age of 65 years and of 1.07 in BRCA1 carriers [4, 9]. The IMPACT study [8] was the first prospective study to demonstrate that men harboring BRCA mutations (especially BRCA2), are at higher risk for PCa. Castro et al. [7] has also demonstrated that germ line BRCA1&2 mutations were associated with higher grade, stage, nodal involvement and metastasis at diagnosis of PCa. There is even suggesting evidence that BRCA mutations result in an increased risk of developing castrate resistant prostate cancer (CRPC) [10].

There is a large proportion of BRCA carriers among Ashkenazi Jews compared to other populations in the world, 2.6% compared to 0.2%, respectively [11]. In our center there is a unique clinic, aimed specifically at male BRCA gene mutation carriers. We offer a comprehensive screening program for BRCA related cancers (prostate [10], male breast, pancreas and melanoma).

Prostate growth is androgen dependent [12] in males, testosterone is synthesized primarily in the testicular Leydig cells. The amount of total testosterone (TT) produced is under the control of luteinizing hormone (LH), secreted by the anterior pituitary, and regulated by the hypothalamic–pituitary–testicular axis. When TT levels are low, gonadotropin-releasing hormone (GnRH) is released by the hypothalamus, which in turn stimulates the pituitary gland to release follicle stimulating hormone (FSH) and LH. These latter two hormones stimulate the testis to synthesize TT. Finally, increasing levels of TT through a negative feedback loop act on the hypothalamus and pituitary to inhibit the release of GnRH and FSH/LH, respectively.

There are published reports demonstrating an association between PCa and TT [13], but its exact mechanism remains quite elusive. The correlation between TT and prostate disease, whether benign or malignant, is due to a myriad of factors. These include various methodologies used for measuring TT, the unknown contribution of the androgen receptor (AR), the effect of chronic TT levels and the discordance between serum and intra-prostatic TT levels [14]. However, other studies have shown that high TT levels do not culminate in higher incidence of PCa [15, 16]. A specific “Saturation model” has also been suggested to explain the seemingly contradictory results of testosterone’s link to PCa [17]. Succinctly, this proposed model demonstrates that TT changes below the point of maximal androgen-AR binding will elicit substantial changes in PCa growth, as seen with castration. However, once maximal androgen-AR binding is reached the presence of additional androgen produces little further effect [17].

There are no published data to date, on the association between TT levels, PCa and BRCA mutation carriers. Therefore, our aim in this study was to characterize TT and additional hormonal levels in male BRCA carriers and try to ascertain if any difference exists in comparison to a group of healthy males without BRCA mutations.

## RESULTS

Overall 87 male carriers and 43 non-carriers were enrolled in our study and both groups were age matched. Only 29 patients (22.33%) refused to participate in our study. A third of the carriers were BRCA2 carriers and the rest were BRCA1. As shown in Table 1 the median age, BMI and comorbidities in both groups were similar. No suspicious findings were reported in the digital rectal examination of patients in both groups.

**Table 1:** Clinical characteristics of patients

	*Carriers*	*Non carriers*	*p Value*
Num. of Patients, *n*	87	43	-
Mean age (IQR)	54.7 (9.2)	53.4 (8.7)	*0.43*
Age group			
40-50, *n* (%)	35 (40.2%)	19 (44.2%)	*0.76*
51-60, *n* (%)	20 (23%)	11 (25.6%)	
61-70, *n* (%)	32 (36.8%)	13 (30.2%)	
Median BMI (IQR)	27.83 (4.58)	27.3 (4.1)	*0.5*
Comorbidities			
NIDDM, %	14.8%	7%	0*.19*
Hypertension, %	29.5%	23.3%	0*.42*
Ischemic heart disease,%	9.1%	7%	0*.66*
Oncologic diseases, %	4.6%	9.5%	*0.27*
BRCA1, *n* (%)	58 (66.67%)	-	
BRCA2, *n* (%)	29 (33.33%)		
Type of BRCA mutation		-	
185delAG, *n* (%)	43 (50%)		
6174deIT, *n* (%)	24 (27.9%)		
5382insc, *n* (%)	10 (11.6%)		
Y978X, *n* (%)	3 (3.5%)		
Others	6 (7%)		

**Figure 1:**
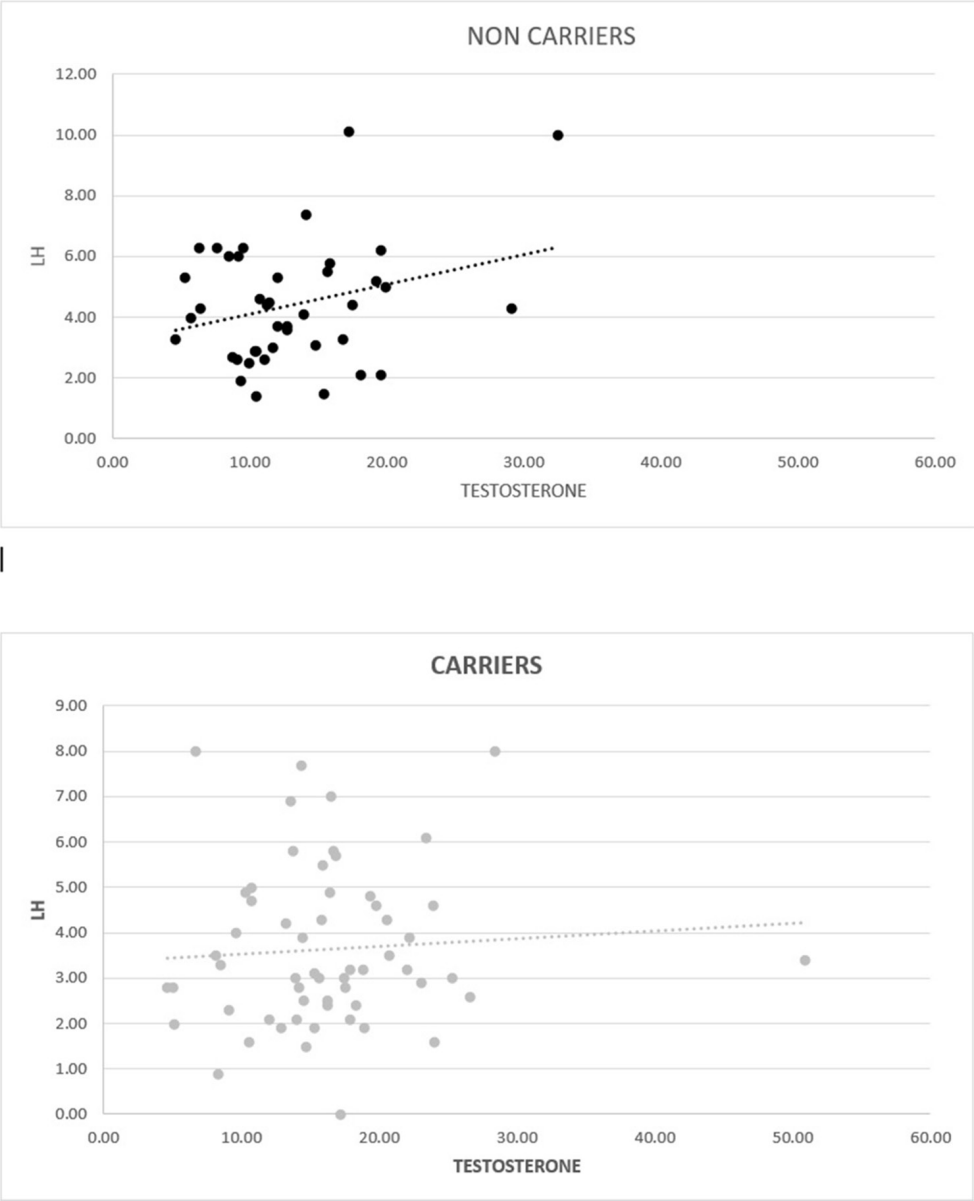
Graphic correlation between testosterone (nmol/l) and LH (IU/ml) in non-carriers (p=0.29) and carriers (p=0.6)

The blood tests shown in Table 2, demonstrate similar FSH and sex hormone binding globulin (SHBG) levels but slightly higher (although not statistically significant) PSA in the non-carrier group. However, a significant statistical difference was noted in the mean TT and free androgen index (FAI) with higher levels in the carrier group, although both groups were in the normal age specific (>40 years)) TT range (6.6-25.3 nmol/l) and FAI range (30-150) [18]. Additionally, mean prolactin levels were significantly lower in the carrier group. In an attempt to discover the factors predicting the high TT levels specifically in the carrier group, a multivariable linear regression analysis (MLRA) was done and compared to the MLRA predicting TT levels in non-carriers (Table 3). As physiologically expected, in the non-carrier group increasing LH and decreasing BMI were both found to be TT predictors. Interestingly however, in the carrier group only decreasing BMI was found to be a predictor. Tests to see if the data met the assumption of collinearity were performed, demonstrating that multicollinearity was not a concern for the MLRAs performed in both groups (Table 3). When controlling for the type of mutation, no difference was noted between BRCA1 and BRCA2 mutation carrying patients. Figure 1 demonstrates the correlation between LH and TT in both groups with visually different trend lines.

**Table 2:** Blood hormonal levels

	*Carriers*	*Non carriers*	*p Value*
Mean PSA ng/ml (SD)	1.7 (1.7)	2.7 (4.4)	*0.065*
*Mean Prolactin ng/ml (SD)*	*6.4 (4.3)*	8.7 (7***)***	*0.048*
Mean FSH IU/l (SD)	5.2 (3)	5.9 (2.9)	0.*239*
Mean LH IU/l (SD)	3.78 (1.97)	4.3 (2)	0.*168*
*Mean Total Testosterone nmol/l (SD)*	16.*7 (7.7)*	13.*5 (6.3)*	0.0*3*
*Mean Free Androgen Index (SD)*	39.*5 (12.9)*	34*.8 (10.8)*	0.0*5*
Mean Sex Hormone Binding Globulin nmol/l (SD)	40.8 (18.9)	41.5 (23.1)	0.8*63*

**Table 3:** Multivariable linear regression analysis predicting total testosterone in BRCA mutation carriers and non-carriers

*Non-carriers*							*Carriers*					
	*p Value* ``	*Odds ratio*	*95% confidence interval*		*Tolerance*	VIF*	*p Value*	*Odds ratio*	*95% confidence interval*		*Tolerance*	VIF*
			*Lower*	*Upper*					*Lower*	*Upper*		
***Age***	.481	-.095	-.365	.176	.575	1.739	*.317*	.153	-.151	.456	.429	2.330
***BMI***	*.001*	*-.793*	*-1.221*	*-.365*	*.917*	*1.091*	*.004*	*-.761*	*-1.261*	*-.261*	.757	1.322
***PSA***	.265	.258	-.206	.723	.720	1.390	*.320*	-.643	-1.926	.641	.619	1.615
***TSH***	.185	.957	-.484	2.398	.867	1.153	*.522*	-.483	-1.987	1.022	.793	1.262
***FSH***	.095	-.771	-1.684	.142	.430	2.323	*.927*	-.037	-.845	.770	.600	1.665
***LH***	*.013*	*1.618*	*.369*	*2.867*	*.474*	*2.111*	*.107*	.952	-.214	2.118	.662	1.511
***Prolactin***	.780	-.037	-.307	.233	.804	1.244	*.644*	-.107	-.568	.355	.889	1.124

*VIF = Variance Inflation Factor.

## DISCUSSION

PCa is the second most common cancer in men worldwide and sixth most common cause of death [19]. Men with germline mutations in BRCA1 or BRCA2 genes have an increased risk of PCa and carriers present at a younger age with a more aggressive disease and with a higher mortality rate [6, 7, 20, 21]. BRCA2 mutation status has been confirmed as an independent prognostic factor for poorer outcome [7].

The results of our hypothesis generating study show that among men between the ages of 40-70, BRCA carriers had a significantly higher plasma levels of TT and FAI while their prolactin levels were lower compared to controls. Furthermore, univariate and MLRA demonstrated that TT level was not correlated to LH levels among BRCA carriers.

This is not a new concept, as other reports have shown that TT secretion maybe independent of LH in certain circumstances. Patients with CRPC have been shown to have an increase in a series of genes mediating androgen synthesis and catabolism in the prostate [22]. This is consistent with (and provides a mechanism for) the substantial levels of TT found in prostate biopsies from CRPC patients compared with normal prostate or non-cancerous prostate after androgen deprivation therapy (ADT) [23, 24]. More importantly, this biologic mechanism explains how PCa adapts to ADT and why available AR antagonists do not have substantial activity against the castrate resistant tumor cells emerging after ADT. It has also been shown that advanced CRPC cells acquire complete steroidogenic ability to synthesize TT and underline the fact that castration and inhibition of TT production in the testes may not achieve castration in PCa cells in advanced stages of the disease [25].

Even in healthy men it has been demonstrated that LH and TT concentrations show significant seasonal variations, and that LH and TT are not in phase, differing in their secretory zenith by about 6 months, thus excluding a hierarchical pivotal role of LH in regulating seasonal variations of TT levels [26]. It was also shown in the same study that FSH and prolactin concentrations had no significant seasonal rhythm and no clear correlation to TT levels, [26, 27]. (as demonstrated in our MLRA results). In fact, it has even been shown that some circadian clock genes do influence fertility and TT seasonality in humans without a correlation to LH levels [28].

Additionally, several experiments performed in animal models have shown an LH independent pathway for TT secretion. These include a macrophage derived factor stimulating TT production by Leydig cells [29], physiological level of lactate causing TT production through an LH independent mechanism involving the increased activities of adenylyl cyclase, cytochrome P450scc, and L-type Ca [2]^+^ channel [30] and GNRH2 autocrine or paracrine regulation of TT secretion in swine testis without LH involvement [31].

The exact mechanism leading to aggressive PCa in BRCA carriers is unknown. Taylor et al [32], recently characterized genomic alterations among BRCA carriers with localized PCa. Their study demonstrated that localized castration-sensitive BRCA2-mutant tumours are uniquely aggressive due to de novo aberration in genes usually associated with metastatic CRPC disease, justifying aggressive initial treatment.

Summing up the last paragraphs, it seems that BRCA carriers with PCa undergo mutations culminating in a very early status of CRPC, where TT has been shown to be secreted in an LH independent manner. We therefore, hypothesized that BRCA carriers, even those without PCa, might have similar mutations causing higher TT to be secreted in an LH independent manner in accordance with our results. The higher TT levels, could help explain their more aggressive early onset PCa. In a meta-analysis of hormonal predictors of PCa, Shaneyfelt et al. have shown that even healthy men who have TT levels in the upper quartile of the population distribution, are 2.34 times more likely to develop PCa [33]. We therefore suggest that there is a correlation between development of PCa in BRCA carriers and hormonal status, specifically through higher TT levels.

There are numerous evidence suggesting a link between hormonal regulation and cancer among BRCA carriers. First, the most common malignancies in BRCA carriers are breast, ovary and prostate, which are all hormonal mediated organs [2-4]. Second, it has been shown that mutations in BRCA1 gene in females are associated with low response to ovarian stimulation, and thus pose a major risk factor for infertility [34]. It was hypothesized that due to the deficiency of DNA repair among BRCA carriers, oocytes may be more prone to DNA damage. Third, high TT levels, together with insulin and markers of insulin resistance have been demonstrated to increase breast and PCa incidence and worsen prognosis, even in men and women without BRCA mutations [35, 36]. Clearly, according to these published reports the fact that we have revealed a hormonal correlation with higher TT and FAI levels in male BRCA mutation carriers, who are more prone to develop aggressive PCa, is not entirely surprising.

This is the first prospective study to our knowledge that attempts to study the implication of male BRCA mutations on TT and various other hormones. Although the involved mechanisms are still unknown, this pilot study does reveal some new insights. The higher TT and FAI might possibly be the hormonal linking factor to the aggressive PCa seen in BRCA carriers. Limitations of this study include its small sample size, lack of validation of hormonal levels over time and no comparison to carriers who developed PCa. We are also limited by the fact that Dihydrotestosterone (DHT) levels were not measured, which could have given us additional information. However, the formation of DHT from TT by the enzyme 5-alpha reductase is a process that occurs only in tissues (prostate, seminal vesicles, skin, hair, etc.) and does not occur in the blood stream. Therefore, blood level measurements of DHT often lead to equivocal or even false-negative results [37, 38], rendering this measurement to have less of an impact on our study.

To conclude, in this hypothesis generating study BRCA carriers have been shown to have a significantly higher TT and FAI levels compared to healthy age matched men. It also demonstrates that in contradiction to non-carriers, LH does not significantly predict TT levels in BRCA carriers. The reasons for these differences are still a mystery and further research is warranted in a larger BRCA carrier cohort including patients with and without PCa. Incorporation of validative measurements of hormonal levels over time would also have a significant impact. In addition, genetic testing assessing the same mutations in Taylor’s et al. [32] study should be sought in BRCA carriers without PCa. Deciphering the biologic mechanism responsible for the increased TT in BRCA carriers could help us get a better understanding why they are more prone to develop aggressive and early onset PCa.

## MATERIALS AND METHODS

After institutional review board approval, BRCA carriers and non-carriers between the ages of 40-70 were prospectively enrolled in our study during the years 2015 – January 2016. All participants provided written informed consent for participation in the study. All study participants were enrolled after testing performed in our unique BRCA male clinic, operated in our tertiary medical center. Participants came to our clinic to be tested for BRCA 1& 2 mutations due to a significant familial cancer history. The testing was done for all known 15 founder mutations in Jews, i.e., ‘‘The Jewish panel’’ [39-42] in BRCA1 (ONIM: 113705) and BRCA2 (ONIM: 600185) genes (Table 4). Rapid sequencing for all BRCA mutations was not done and testing was only done for these specific founder mutations, due to the fact that they constitute 95% of all mutations in Jews. The search for mutations was done by Nano-Fluidic Chip 96.96CS (Genotyping Dynamic array IFC) using the Biomark HD system™ by Fluidigm.

**Table 4:** 15 known BRCA 1&2 founder mutations in Jews

BRCA mutations tested	
BRCA1: NM_007294.2	Known clinical name (According to GenBank U14680.1)
p.E23VfsX17, c.68_69delAG	185delAG
p.Q1756PfsX74, c.5266dupC	5382insC
p.E1346KfsX20, c.4035delA	4153delA
p.A1708E, c.5123C>A	A1708E
P.Y978X, c.2934T>G	Y978X
p.C61G, C.181T>G	300T>G
P.C328X, c.981delAT	llOOdelAT
p.E720X, c.2158G>T	E720X
p.W1508X, c.4524G>A	W1508X

BRCA2: NM_000059.3	Known clinical name (According to GenBank U43746)
p.S1982RfsX22, c.5946delT	6174delT
p.E2846GfsX22, c.8537_8538delAG	8765delAG
p.V1283KfsX2, c.3847_3848delGT	4075delGT
c.67+lG>A	IVS2+1G>A
p.R2336P, c.7007G>C	R2336P
p.E1646QfsX23, c.4936_4939delGAAA	5164del4

This table is based on data from the Cancer Information Core Database.

http://research.nhgri.nih.gov/projects/bic/

Inclusion criteria included males between 40-70 who were genetically confirmed to be BRCA carriers or non-carriers and able and willing to sign an informed consent. The exclusion criteria included any history of PCa therapy or BPH surgery and any endocrinologic disease, due to its possible effect on the hormonal status.

Medical comorbidities of all patients were recorded and blood tests were taken, including PSA, TT, FSH, LH, FAI, SHBG and prolactin. Blood samples were collected between 0800 AM and 1100 AM to minimize the confounding effects of diurnal variation in the concentration levels of the hormones. The levels of PSA, TT, LH, FSH and prolactin, were measured by radioimmunoassay. All results were analyzed and compared between both groups.

Statistical analysis included descriptive analysis (range, mean, and median), standard deviation for continuous variables, proportions for discrete variables, and comparative tests (chi-square for discrete variables and Mann-Whitney for continuous variables). MLRA were done to compare predictors of hormonal levels among carriers and non-carriers. Furthermore, tests were performed to assess if the data in the MLRA met the assumption of collinearity. All analyses were calculated using the SPSS software version 23.0 (SPSS Inc., Chicago, IL). All statistical tests were two - tailed and a p value of 0.05 was considered statistically significant.

## Abbreviations

ADT: Androgen deprivation therapy
AR: Androgen receptor
BRCA: Breast cancer susceptibility genes
CRPC: Castrate resistant prostate cancer
DHT: Dihydrotestosterone
FAI: Free androgen index
FSH: Follicle stimulating hormone
GnRH: Gonadotropin-releasing hormone
LH: Luteinizing hormone
MLRA: Multivariate logistic regression analysis
PCa: Prostate cancer
PSA: Prostate specific antigen
SHBG: Sex hormone binding globulin
TT: Total testosterone

## References

[1] Yoshida K, Miki Y (2004). Role of BRCA1 and BRCA2 as regulators of DNA repair, transcription, and cell cycle in response to DNA damage. Cancer Sci.

[2] King MC, Marks JH, Mandell JB (2003). Breast and ovarian cancer risks due to inherited mutations in BRCA1 and BRCA2. Science.

[3] Ford D, Easton DF, Bishop DT, Narod SA, Goldgar DE (1994). Risks of cancer in BRCA1-mutation carriers. Breast Cancer Linkage Consortium. Lancet.

[4] Thompson D, Easton D (2001). Variation in cancer risks, by mutation position, in BRCA2 mutation carriers. Am J Hum Genet.

[5] Mitra AV, Bancroft EK, Barbachano Y, Page EC, Foster CS, Jameson C, Mitchell G, Lindeman GJ, Stapleton A, Suthers G, Evans DG, Cruger D, Blanco I (2011). Targeted prostate cancer screening in men with mutations in BRCA1 and BRCA2 detects aggressive prostate cancer: preliminary analysis of the results of the IMPACT study. BJU Int.

[6] Tryggvadottir L, Vidarsdottir L, Thorgeirsson T, Jonasson JG, Olafsdóttir EJ, Olafsdóttir GH, Rafnar T, Thorlacius S, Jonsson E, Eyfjord JE, Tulinius H (2007). Prostate cancer progression and survival in BRCA2 mutation carriers. J Natl Cancer Inst.

[7] Castro E, Goh C, Olmos D, Saunders E, Leongamornlert D, Tymrakiewicz M, Mahmud N, Dadaev T, Govindasami K, Guy M, Sawyer E, Wilkinson R, Ardern-Jones A (2013). Germline BRCA mutations are associated with higher risk of nodal involvement, distant metastasis, and poor survival outcomes in prostate cancer. J Clin Oncol.

[8] Bancroft EK, Page EC, Castro E, Lilja H, Vickers A, Sjoberg D, Assel M, Foster CS, Mitchell G, Drew K, Mæhle L, Axcrona K, Evans DG (2014). Targeted prostate cancer screening in BRCA1 and BRCA2 mutation carriers: results from the initial screening round of the IMPACT study. Eur Urol.

[9] Thompson D, Easton DF (2002). Cancer incidence in BRCA1 mutation carriers. J Natl Cancer Inst.

[10] Helleday T (2016). PARP inhibitor receives FDA breakthrough therapy designation in castration resistant prostate cancer: beyond germline BRCA mutations. Ann Oncol.

[11] Giusti RM, Rutter JL, Duray PH, Freedman LS, Konichezky M, Fisher-Fischbein J, Greene MH, Maslansky B, Fischbein A, Gruber SB, Rennert G, Ronchetti RD, Hewitt SM (2003). A twofold increase in BRCA mutation related prostate cancer among Ashkenazi Israelis is not associated with distinctive histopathology. J Med Genet.

[12] Jin B, Conway AJ, Handelsman DJ (2001). Effects of androgen deficiency and replacement on prostate zonal volumes. Clin Endocrinol.

[13] Michaud JE, Billups KL, Partin AW (2015). Testosterone and prostate cancer: an evidence-based review of pathogenesis and oncologic risk. Ther Adv Urol.

[14] Loughlin KR (2016). The testosterone conundrum: the putative relationship between testosterone levels and prostate cancer. Urol Oncol.

[15] Andriole GL, Bostwick DG, Brawley OW, Gomella LG, Marberger M, Montorsi F, Pettaway CA, Tammela TL, Teloken C, Tindall DJ, Somerville MC, Wilson TH, Fowler IL (2010). Effect of dutasteride on the risk of prostate cancer. N Engl J Med.

[16] Hoque A, Yao S, Till C, Kristal AR, Goodman PJ, Hsing AW, Tangen CM, Platz EA, Stanczyk FZ, Reichardt JK, vanBokhoven A, Neuhouser ML, Santella RM (2015). Effect of finasteride on serum androstenedione and risk of prostate cancer within the prostate cancer prevention trial: differential effect on high- and low-grade disease. Urology.

[17] Morgentaler A, Traish AM (2009). Shifting the paradigm of testosterone and prostate cancer: the saturation model and the limits of androgen-dependent growth. Eur Urol.

[18] Kelsey TW, Li LQ, Mitchell RT, Whelan A, Anderson RA, Wallace WH (2014). A validated age-related normative model for male total testosterone shows increasing variance but no decline after age 40 years. PLoS One.

[19] Jemal A, Bray F, Center MM, Ferlay J, Ward E, Forman D (2011). Global cancer statistics. CA Cancer J Clin.

[20] Edwards SM, Evans DG, Hope Q, Norman AR, Barbachano Y, Bullock S, Kote-Jarai Z, Meitz J, Falconer A, Osin P, Fisher C, Guy M, Jhavar SG (2010). Prostate cancer in BRCA2 germline mutation carriers is associated with poorer prognosis. Br J Cancer.

[21] Mitra A, Fisher C, Foster CS, Jameson C, Barbachanno Y, Bartlett J, Bancroft E, Doherty R, Kote-Jarai Z, Peock S, Easton D, Eeles R, IMPACT and EMBRACE Collaborators (2008). Prostate cancer in male BRCA1 and BRCA2 mutation carriers has a more aggressive phenotype. Br J Cancer.

[22] Stanbrough M, Bubley GJ, Ross K, Golub TR, Rubin MA, Penning TM, Febbo PG, Balk SP (2006). Increased expression of genes converting adrenal androgens to testosterone in androgen-independent prostate cancer. Cancer Res.

[23] Mohler JL, Gregory CW, Ford OH, Kim D, Weaver CM, Petrusz P, Wilson EM, French FS (2004). The androgen axis in recurrent prostate cancer. Clin Cancer Res.

[24] Nishiyama T, Hashimoto Y, Takahashi K (2004). The influence of androgen deprivation therapy on dihydrotestosterone levels in the prostatic tissue of patients with prostate cancer. Clin Cancer Res.

[25] Dillard PR, Lin MF, Khan SA (2008). Androgen-independent prostate cancer cells acquire the complete steroidogenic potential of synthesizing testosterone from cholesterol. Mol Cell Endocrinol.

[26] Bellastella G, Pane E, Iorio S, De Bellis A, Sinisi AA (2013). Seasonal variations of plasma gonadotropin, prolactin, and testosterone levels in primary and secondary hypogonadism: evidence for an independent testicular role. J Endocrinol Invest.

[27] Maes M, Mommen K, Hendrickx D, Peeters D, D'Hondt P, Ranjan R, De Meyer F, Scharpé S (1997). Components of biological variation, including seasonality, in blood concentrations of TSH, TT3, FT4, PRL, cortisol and testosterone in healthy volunteers. Clin Endocrinol.

[28] Kovanen L, Saarikoski ST, Aromaa A, Lonnqvist J, Partonen T (2010). ARNTL (BMAL1) and NPAS2 gene variants contribute to fertility and seasonality. PLoS One.

[29] Lukyanenko YO, Carpenter AM, Brigham DE, Stocco DM, Hutson JC (1998). Regulation of Leydig cells through a steroidogenic acute regulatory protein-independent pathway by a lipophilic factor from macrophages. J Endocrinol.

[30] Lin H, Wang SW, Wang RY, Wang PS (2001). Stimulatory effect of lactate on testosterone production by rat Leydig cells. J Cell Biochem.

[31] Desaulniers AT, Cederberg RA, Mills GA, Ford JJ, Lents CA, White BR (2015). LH-independent testosterone secretion is mediated by the interaction between GNRH2 and its receptor within porcine testes. Biol Reprod.

[32] Taylor RA, Fraser M, Livingstone J (2017). Germline BRCA2 mutations drive prostate cancers with distinct evolutionary trajectories. Nat Commun.

[33] Shaneyfelt T, Husein R, Bubley G, Mantzoros CS (2000). Hormonal predictors of prostate cancer: a meta-analysis. J Clin Oncol.

[34] Oktay K, Kim JY, Barad D, Babayev SN (2010). Association of BRCA1 mutations with occult primary ovarian insufficiency: a possible explanation for the link between infertility and breast/ovarian cancer risks. J Clin Oncol.

[35] Pasanisi P, Villarini A, Bruno E, Raimondi M, Gargano G, Berrino F (2010). Nutritional advice to breast cancer survivors. Support Care Cancer.

[36] Parikesit D, Mochtar CA, Umbas R, Hamid AR (2016). The impact of obesity towards prostate diseases. Prostate Int.

[37] Walter KN, Kienzle FB, Frankenschmidt A, Hiort O, Wudy SA, van der Werf-Grohmann N, Superti-Furga A, Schwab KO (2010). Difficulties in diagnosis and treatment of 5alpha-reductase type 2 deficiency in a newborn with 46, XY DSD. Horm Res Paediatr.

[38] Maimoun L, Philibert P, Cammas B, Audran F, Bouchard P, Fenichel P, Cartigny M, Pienkowski C, Polak M, Skordis N, Mazen I, Ocal G, Berberoglu M (2011). Phenotypical, biological, and molecular heterogeneity of 5alpha-reductase deficiency: an extensive international experience of 55 patients. J Clin Endocrinol Metab.

[39] Abeliovich D, Kaduri L, Lerer I, Weinberg N, Amir G, Sagi M, Zlotogora J, Heching N, Peretz T (1997). The founder mutations 185delAG and 5382insC in BRCA1 and 6174delT in BRCA2 appear in 60% of ovarian cancer and 30% of early-onset breast cancer patients among Ashkenazi women. Am J Hum Genet.

[40] Lerer I, Wang T, Peretz T, Sagi M, Kaduri L, Orr-Urtreger A, Stadler J, Gutman H, Abeliovich D (1998). The 8765delAG mutation in BRCA2 is common among Jews of Yemenite extraction. Am J Hum Genet.

[41] Shiri-Sverdlov R, Gershoni-Baruch R, Ichezkel-Hirsch G, Gotlieb WH, Bruchim Bar-Sade R, Chetrit A, Rizel S, Modan B, Friedman E (2001). The Tyr978X BRCA1 mutation in non-Ashkenazi Jews: occurrence in high-risk families, general population and unselected ovarian cancer patients. Community Genet.

[42] Sagi M, Eilat A, Ben Avi L, Goldberg Y, Bercovich D, Hamburger T, Peretz T, Lerer I (2011). Two BRCA1/2 founder mutations in Jews of Sephardic origin. Fam Cancer.

